# Supplementary material for: Identification of key genes associated with idiopathic pulmonary fibrosis and sarcopenia by bioinformatics analysis
Source: PLoS One. 2025 Dec 1;20(12):e0335067. doi: 10.1371/journal.pone.0335067 (PMC12668514; doi:10.1371/journal.pone.0335067)
Supplement: Attachment — Submitted filename: Response to Reviewers.docx [file pone.0335067.s002.docx]

We would like to express our sincere gratitude to you and the reviewers for your valuable comments on our manuscript. In light of the thorough and insightful feedback provided, we have revised the manuscript accordingly. We have added relevant content to address the reviewers' suggestions, and we have rewritten several sections to ensure clarity and completeness. We hope that these modifications meet the reviewers' expectations and enhance the overall quality of the manuscript.

Reviewer #1: This study integrates multiple public databases and bioinformatics approaches to explore the shared molecular mechanisms underlying idiopathic pulmonary fibrosis (IPF) and sarcopenia, with particular emphasis on endoplasmic reticulum stress-related crosstalk genes (ERSRCGs) and the development of potential diagnostic models. The methodology incorporates differential expression analysis, WGCNA, GSVA/GSEA, immune infiltration profiling, protein–protein interaction (PPI) network construction, and regulatory network analysis, forming a comprehensive workflow with a degree of novelty. However, the manuscript still presents several issues that warrant attention and revision:

Consideration 1:

The IPF training set (GSE24206) comprises 17 samples from 11 IPF patients (including paired upper- and lower-lobe samples from 6 patients and single samples from 5 patients) along with 6 control specimens obtained from healthy donor lungs during lung volume reduction surgery at transplantation. Given the relatively small sample size of the IPF training set, it is recommended that the authors augment the dataset by incorporating additional publicly available cohorts to strengthen the statistical power of their analysis. Public repositories are valuable resources for hypothesis generation and gene discovery; however, validation in larger, clinically stratified patient cohorts would substantially enhance the translational potential and reliability of these findings.

RE 1: Thank you for your careful review and valuable comments.

In this study, we selected publicly available datasets with clear clinical annotations and included all eligible samples, resulting in 17 IPF cases and 6 controls in the training set. The sample size reflects both the strict inclusion criteria and the challenges of obtaining well-annotated clinical samples for IPF research. Due to research constraints and timelines, we chose not to merge additional datasets. Nevertheless, through reasonable statistical design and rigorous batch effect correction, our data analysis results retain scientific significance and interpretability, providing valuable insights into potential mechanisms. We acknowledge that a larger cohort would further strengthen our conclusions, and we plan to expand the sample size and validate findings in future studies.

Once again, thank you for your valuable suggestions.

Consideration 2:

The manuscript is lengthy; therefore, it is recommended to condense the description of the Methods section for improved clarity and conciseness.

RE 2: Thank you for your careful review and valuable comments.

Based on your suggestions, we will further refine and optimize the structure of the Methods section to highlight the main analytical workflow, simplify the description of technical details, and move some detailed parameters and database information to the supplementary materials to improve clarity and readability. Specific revisions are detailed on page 4-10, lines 96-288.

Thank you again for your valuable suggestions.

Consideration 3:

The manuscript requires careful revision to refine the language and improve overall readability.

RE 3: Thank you for your attention to the language and readability of the manuscript. In response to this valuable feedback, we have commissioned a professional language editing service to revise the entire manuscript comprehensively and obtained a proof of editing to ensure that the manuscript meets international academic standards. We believe that these efforts have greatly improved the quality and readability of the manuscript, and we appreciate your suggestions, which have helped us further enhance our work.

Reviewer #2: Generally speaking, these data and analyses support the conclusions. Here my suggestions:

1.In methods, the authors expressed that IPFRDEGs were defined as adj. p < 0.05 and |logFC| > 0.0; the genes with adj. p < 0.05 and logFC > 0.0 were regarded as up-regulated, and genes with adj. p < 0.05 and logFC < 0.0 were regarded as down-regulated? Usually, differentially expressed genes (DEGs) were considered as P value <0.05 and |log fold change (FC)| > 1, in some times, for example, in cytokine differential expression analysis, a cytokine with fold change (FC) ≥ 1.2 or < 0.83 and a p-value <0.05 was considered statistically differentially expressed. Taken together, It’s not common that the threshold value selected in this paper, is there any supported robust evidence be cited? Please kindly clarify and explain.

RE 1: Thank you for your interest in our research.

We chose |logFC| > 0.0 mainly to include as many differentially expressed genes as possible in the initial screening phase, to capture subtle changes that may have biological significance for the progression of IPF and Sarcopenia. This strategy is particularly suitable for exploratory research, as it can more comprehensively identify potential key genes and pathways involved in disease mechanisms (PMID: 34786719). By combining this allowed fold change threshold with strict statistical significance criteria (adjusted p < 0.05), we aim to minimize the risk of missing important molecular signals, especially considering the complexity and heterogeneity of these diseases. We believe this approach is reasonable for hypothesis generation and comprehensive bioinformatics analysis.

Thank you again for your valuable suggestions.

2. The analyzed IPF-related datasets GSE24206 and GSE53845, Sarcopenia-related datasets GSE8479 and GSE1428 obtained from the GEO database were relatively old (more than 10 years), is there any new datasets available within 5 years?

RE 2: Thank you for your detailed review and valuable comments.

Although the dataset we have chosen was released earlier, it remains the most authoritative, frequently cited, highest quality, and widely validated classic cohort, still extensively used in this field. Furthermore, the innovation of this study mainly lies in the combination of ERS-related genes, comprehensively utilizing WGCNA, GSVA, LASSO, and immune infiltration analysis methods to reveal the common molecular mechanisms and potential diagnostic markers of IPF and sarcopenia, rather than solely relying on the age of the data. Based on this, we believe that the existing dataset still has representativeness and scientific value.

Thank you again for your valuable suggestions.

3. I suggest move some contents to supplementary materials. There exist too many words and figures, and it’s too difficult for readers to grasp main findings. For example, the results of GSVA analyses should be modified.

RE 3: Thank you for your thorough review and valuable comments.

We will revise the manuscript based on your suggestions, streamlining some results and related content to retain only the core results and representative figures closely related to the main conclusions, while moving the remaining detailed analyses and complete data to the supplementary materials, allowing readers to better grasp the main scientific findings. For specific modifications, please refer to page 20-21, line 551-592.

Thank you again for your valuable suggestions.

4. The English writing should be checked and polished, throughout the whole manuscript.

RE 4: Thank you for your attention to the language and readability of the manuscript. In response to this valuable feedback, we have commissioned a professional language editing service to comprehensively revise the entire manuscript and obtain a proof of editing to ensure it meets international academic standards. We believe these efforts have greatly enhanced the quality and readability of the manuscript, and we appreciate your suggestions, which have helped us further improve our work.
