# Supplementary material for: Identification of key genes associated with idiopathic pulmonary fibrosis and sarcopenia by bioinformatics analysis
Source: PLoS One. 2025 Dec 1;20(12):e0335067. doi: 10.1371/journal.pone.0335067 (PMC12668514; doi:10.1371/journal.pone.0335067)
Supplement: Attachment — Submitted filename: Response_to_Reviewers_auresp_2.docx [file pone.0335067.s003.docx]

We would like to express our sincere gratitude to you and the reviewers for your valuable comments on our manuscript. In light of the thorough and insightful feedback provided, we have revised the manuscript accordingly. We have added relevant content to address the reviewers' suggestions, and we have rewritten several sections to ensure clarity and completeness. We hope that these modifications meet the reviewers' expectations and enhance the overall quality of the manuscript.

Reviewer #1:

1. This study employed the criteria of adj. p 0 to define differentially expressed genes. This threshold is relatively lenient, which may result in a larger number of genes being included, thereby increasing the risk of false positives. Although the authors explain that this approach is intended to explore potential signals, it is still recommended to provide a more detailed rationale in the Methods section and to emphasize its limitations in the Discussion. Additionally, results using a stricter threshold (e.g., |logFC| > 1) can be provided in the Supplementary materials as a control to enhance the robustness of the conclusions.

RE 1：Thank you for your careful review and valuable comments.

In the previous reply, we also mentioned that the reason for choosing this standard is mainly because the positioning of this study is exploratory analysis, and the sample size is relatively limited. If overly strict fold change thresholds are adopted in the initial screening phase, it may overlook some genes that have potential biological significance in the occurrence and development of diseases but have relatively subtle expression differences. In order to capture potential signals as comprehensively as possible, we chose |logFC| > 0 combined with strict statistical standards (adj.p < 0.05) to improve the sensitivity of the screening. We emphasized the limitations of this strategy in the discussion section . Specific revisions are detailed on page 33, lines 910-922. Based on the exploratory nature of the study and the scale of the data, we believe that the current analysis framework is more suitable for the objectives of this study, and therefore, we did not add parallel analyses with stricter thresholds. However, we plan to conduct supplementary analyses with stricter thresholds in the future.

Thank you again for your valuable suggestions.

2. The authors successfully utilized CIBERSORT to reveal changes and correlations in the immune cell composition of IPF and sarcopenia samples; however, the discussion section remains at a descriptive level. There is a lack of in-depth interpretation of the biological significance of these findings. For instance, the study found a strong correlation between resting mast cells and activated NK cells, which is an intriguing discovery, but the functional implications of this correlation within the context of IPF (whether it promotes fibrosis or serves a protective mechanism) have not been explored.

RE 2：Thank you for your careful review and valuable comments.

We will further supplement the functional significance of the immune cell interaction results in the revised manuscript. Specific revisions are detailed on page 30 -32, lines 847- 880.

Thank you again for your valuable suggestions.

3. The ultimate goal of the research is to discover biomarkers that can be used for diagnosis or treatment. Although diagnostic models have been constructed, the discussion section fails to adequately articulate their potential clinical value. The model performed poorly in an independent validation cohort (AUC ~0.56), which is a significant limitation; however, the authors merely state the facts without analyzing the reasons (such as batch effects or disease heterogeneity) or exploring whether there are still specific application scenarios (such as screening in high-risk populations rather than for definitive diagnosis).

RE 3：Thank you for your careful review and valuable comments.

Thank you for your detailed review and valuable comments. Based on your suggestions, we will include an analysis of the reasons for the poor performance of external validation in the revised manuscript and further elaborate on the value of the model in potential clinical application scenarios, while emphasizing its significant importance in revealing the molecular mechanisms of IPF and sarcopenia, to enhance the completeness and translational relevance of the discussion. Specific revisions are detailed on page 32, lines 898 - 905.

Thank you again for your valuable suggestions.

4. This study is purely a bioinformatics analysis and lacks in vitro or in vivo experiments to validate the functions of key genes such as CTH and FOXO1. It is recommended to clearly state this limitation in the discussion and suggest that future research verify the specific mechanisms of these genes in IPF and sarcopenia through gene knockdown/overexpression experiments, animal models, or clinical samples.

RE 4：Thank you for your careful review and valuable comments.

As you pointed out, this study belongs to pure bioinformatics analysis, lacking in vivo and in vitro experimental validation, which we have noted as a limitation in the discussion section. Based on your suggestion, we will further elaborate on the characteristics of the current lack of in vivo and in vitro experimental validation in the discussion section and propose that future research will explore the mechanisms of genes such as *CTH* and *FOXO1* through gene functional experiments, animal models, and clinical sample studies. For specific modifications, please refer to page 33, lines 915 - 922.

Thank you again for your valuable suggestions.

5. Any research inevitably encounters results that do not fully align with expectations, and openly discussing these aspects reflects scientific rigor. For instance, the expression changes of ER stress-related genes in the two diseases are not entirely consistent; the pathways enriched by GSEA/GSVA are quite broad, with some showing only a weak direct association with ER stress. The authors have chosen to emphasize results that support their hypothesis while avoiding these complexities.

RE 5：Thank you for your careful review and valuable comments.

We agree with your point that there are indeed some results in the research process that do not completely align with expectations. We will supplement this explanation in the discussion section, emphasizing that these results reflect the complexity of disease mechanisms and the diversity of ER stress effects, and point out their possible research significance. We believe that acknowledging these differences and providing explanations will help enhance the scientific rigor of the research and offer new ideas for subsequent studies. Specific revisions are detailed on page 17, lines 458 - 460, page 28, lines 784 - 790.

Thank you again for your valuable suggestions.

6. The heatmaps and associated bubble charts in Figures 5, 14, and 15 exhibit low color differentiation, and the descriptions of the meanings represented by colors in the figure legends are not sufficiently clear (for instance, the use of non-standard color names such as 'vine purple'). It is recommended to utilize a color scale with higher contrast and to explicitly state the statistical values or grouping information represented by each color in the figure legends.

RE 6：

We greatly appreciate your suggestions regarding the visualization of the charts.

The color scheme currently used in the manuscript was selected after careful consideration of scientific validity and overall aesthetics. The existing color palette clearly distinguishes the direction and intensity of correlations, and the gradient of colors itself is also an important form of visualization. We believe that there are no substantial issues with the current color differentiation, and readers can accurately understand the results by referring to the legend and numerical ranges. Furthermore, we have clearly labeled the grouping information (such as the color distinction between the disease group and the control group) and statistical significance in the legend, so the existing charts already possess sufficient explanatory power and readability. Considering scientific validity, consistency, and aesthetics, we believe that no further modifications to the color scheme or legend in the charts are necessary. Thank you again for your valuable feedback.
